# Polyphosphate Functions *In Vivo* as an Iron Chelator and Fenton Reaction Inhibitor

**DOI:** 10.1128/mBio.01017-20

**Published:** 2020-07-28

**Authors:** François Beaufay, Ellen Quarles, Allison Franz, Olivia Katamanin, Wei-Yun Wholey, Ursula Jakob

**Affiliations:** a Department of Molecular, Cellular and Developmental Biology, University of Michigan, Ann Arbor, Michigan, USA; b Department of Biological Chemistry, University of Michigan, Ann Arbor, Michigan, USA; Yale School of Medicine

**Keywords:** chelator, cisplatin, iron regulation, oxidative damage, polyphosphate, stress response

## Abstract

Maintaining cellular iron homeostasis is critical for organismal survival. Whereas iron depletion negatively affects the many metabolic pathways that depend on the activity of iron-containing enzymes, any excess of iron can cause the rapid formation of highly toxic reactive oxygen species (ROS) through Fenton chemistry. Although several cellular iron chelators have been identified, little is known about if and how organisms can prevent the Fenton reaction. By studying the effects of cisplatin, a commonly used anticancer drug and effective antimicrobial, we discovered that cisplatin elicits severe iron stress and oxidative DNA damage in bacteria. We found that both of these effects are successfully prevented by polyphosphate (polyP), an abundant polymer consisting solely of covalently linked inorganic phosphates. Subsequent *in vitro* and *in vivo* studies revealed that polyP provides a crucial iron reservoir under nonstress conditions and effectively complexes free iron and blocks ROS formation during iron stress. These results demonstrate that polyP, a universally conserved biomolecule, plays a hitherto unrecognized role as an iron chelator and an inhibitor of the Fenton reaction.

## Abstract (precis)

How do organisms deal with free iron? On the one hand, iron is an essential metal that plays crucial structural and functional roles in many organisms. On the other hand, free iron is extremely toxic, particularly under aerobic conditions, where iron rapidly undergoes the Fenton reaction and produces highly reactive hydroxyl radicals. Our study now demonstrates that we have discovered one of the first physiologically relevant nonproteinaceous iron chelators and Fenton inhibitors. We found that polyphosphate, a highly conserved and ubiquitous inorganic polyanion, chelates iron and, through its multivalency, prevents the interaction of iron with peroxide and therefore the formation of hydroxyl radicals. We show that polyP provides a crucial iron reservoir for metalloproteins under nonstress conditions and effectively chelates free iron during iron stress. Importantly, polyP is present in all cells and organisms and hence is likely to take on this crucial function in both prokaryotic and eukaryotic cells.

## INTRODUCTION

Cisplatin [*cis*-diaminedichloroplatinum(II)], originally identified as an inducer of bacterial filamentation, is one of the most widely used drugs in cancer treatment (1, 2). Early mechanistic studies suggested that cisplatin elicits cytotoxicity by acting as a DNA-damaging agent, preferentially cross-linking neighboring purines (3–5). More recent studies, however, revealed that cisplatin also induces cell death in denucleated cells by causing mitochondrial and endoplasmic reticulum stress (6, 7). Indeed, only a small fraction of the intracellular cisplatin pool appears to reach the nucleus, whereas the vast majority binds to the sulfur-containing side chains in proteins as well as to thiol-containing compounds (8–11). Studies in mouse models and ovarian cancer cell lines revealed that tumorous cells gain resistance against cisplatin by increasing their levels of cysteine-enriched peptides (i.e., glutathione) and proteins (i.e., metallothioneins), which capture cisplatin before it reaches the DNA (12–14). This cellular response also seems to aid in mitigating oxidative stress, a frequently observed side effect of cisplatin treatment (15).

Recent studies from our lab revealed that upon cisplatin treatment, cancer cells drastically upregulate and redistribute their levels of inorganic polyphosphate (polyP) (16). PolyP is a polymer of up to 1,000 inorganic phosphate (P_i_) molecules, linked by high-energy phosphoanhydride bonds (reviewed in references 17). In bacteria, polyP protects against a variety of different stress conditions (i.e., oxidative stress, heat stress), stimulates biofilm formation, and regulates virulence (18, 19). Some of these functions can be explained by the ability of polyP to work as a protein-stabilizing scaffold (19). As such, polyP protects soluble proteins against stress-induced aggregation while promoting the formation of functional amyloids, including those involved in biofilms (20). Other potential functions that have been associated with polyP are based on its chemical features as a buffer or high-energy storage molecule (reviewed in reference 21).

To gain more insights into the working mechanism of cisplatin and the role that polyP might play in the cellular response to this drug, we compared the effects of cisplatin treatment on wild-type and polyP-deficient Escherichia coli. Our studies demonstrated that cisplatin triggers a gene expression pattern in wild-type bacteria that is consistent with the inactivation of the repressor Fur, the master regulator of iron homeostasis (22). The resulting gene expression changes lead to an apparent increase in iron uptake, whose deleterious effect is effectively mitigated by endogenous polyP. Deletion of the polyP-synthesizing machinery causes a dramatic increase in cisplatin-induced mutagenesis rate and cell death. Both phenotypes are fully prevented in polyP-depleted bacteria by overexpressing iron storage proteins or by globally reducing the number of iron-containing proteins. Subsequent *in vivo* and *in vitro* studies revealed that polyP acts as a hitherto unknown iron-storage molecule under both stress and nonstress conditions and, by chelating labile iron, acts as a physiologically relevant Fenton inhibitor.

## RESULTS

### PolyP protects E. coli against cisplatin toxicity.

**FIG 1:**
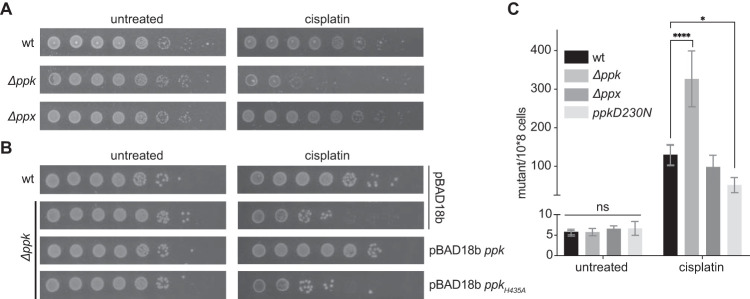
PolyP confers cisplatin resistance in E. coli. (A) A logarithmically growing E. coli MG1655 wild-type (wt), *Δppk*, or *Δppx* strain was 10-fold serially diluted, spotted onto M9-G plates containing 4 μg/ml cisplatin, and incubated at 37°C overnight. (B) Logarithmically growing E. coli wild type containing the empty vector pBAD18b, or the *ppk* mutant strain containing either the empty vector or expressing wild-type PPK or the enzymatically inactive PPK_H435A_ mutant, was 10-fold serially diluted, spotted onto plates containing 0.02% (wt/vol) arabinose and 4 μg/ml cisplatin, and incubated at 37°C overnight. Experiments shown in panels A and B were conducted at least 4 times, and a representative result is shown. (C) The mutagenesis rate of a logarithmically growing wild-type (wt), *Δppk*, or *Δppx* strain or a *Δppk* strain expressing the highly active PPK-D230N mutant protein before and 1 h after treatment with 10 μg/ml cisplatin in liquid culture was determined by counting the number of colonies able to grow on rifampin plates (*n* = 3; *, *P* < 0.05; ****, *P* < 0.0001; ns, nonsignificant, one-way ANOVA).

Despite its prevalent use in antitumor treatment and its known broad antibacterial activity, the exact mechanism by which cisplatin kills cells is still not fully understood. To obtain more detailed insight into the cellular effects of cisplatin, we investigated the responses to and defenses against cisplatin toxicity in bacteria. Based on our recent discovery that polyP serves as an active defense mechanism against oxidative protein damage in bacteria (19), we compared the cisplatin sensitivity of wild-type E. coli with mutant strains that lack either the polyP-synthesizing polyP kinase (PPK) or the polyP-hydrolyzing enzyme exopolyphosphatase (PPX). Whereas the *ppk* deletion strain is unable to make polyP, the *ppx* deletion strain accumulates slightly higher polyP levels than wild-type E. coli under normal growth conditions, and significantly higher polyP levels than wild type under both nutrient shift and oxidative stress conditions (19, 23). We grew all three strains to mid-log phase in minimal morpholinepropanesulfonic acid (MOPS) glucose (MOPS-G) medium and exposed them to increasing amounts of cisplatin either on plates (Fig. 1A) or in liquid culture (see Fig. S1A in the supplemental material). Whereas E. coli wild type or the *ppx* deletion mutant strain did not show any growth defects when incubated on plates supplemented with 4 μg/ml of cisplatin, the *ppk* deletion mutant, which lacks detectable polyP levels (19), showed a reproducible 3- to 4-log reduction in cell survival (Fig. 1A). We obtained very similar results when we conducted the experiments in liquid culture. After a 20-h treatment with 10 μg/ml cisplatin in liquid medium, wild-type E. coli showed an about 3-log decrease in survival while the *ppk* deletion strain showed a greater than 6-log decrease (Fig. S1A).

10.1128/mBio.01017-20.2 FIG S1 Effects of cisplatin treatment of wild-type and polyP-deficient strains on growth in liquid culture, polyP levels, and stress gene expression. (A) E. coli wild-type and *Δppk* strains were grown in MOPS-G to mid-log phase, treated with cisplatin (10 μg/ml) for 20 h in liquid culture, serially diluted, plated onto LB plates, and incubated at 37°C overnight. (B) PolyP from wild-type, *Δppk*, and *Δppx* strains was extracted following a 1-h treatment with 10 μg/ml cisplatin. Change in polyP levels upon a 3-h nutrient shift treatment was used as positive control. (C) Expression of select stress genes in wild-type, *Δppk*, and *Δppx* cells was quantified by RT-PCR after a 15-min treatment with 20 μg/ml cisplatin in MOPS-G medium. Download FIG S1, TIF file, 1.0 MB. Copyright © 2020 Beaufay et al. 2020 Beaufay et al. This content is distributed under the terms of the Creative Commons Attribution 4.0 International license.

Analysis of the endogenous polyP levels in wild-type E. coli did not reveal any significant upregulation of polyP in response to cisplatin treatment (Fig. S1B), suggesting that the steady-state levels of polyP that are present in stationary-phase E. coli (24) are sufficient to confer the observed protection. To ascertain, however, that polyP synthesis is indeed required for the observed cisplatin resistance in wild-type E. coli, we transformed the *ppk* deletion strain with plasmids encoding either the native PPK protein or the previously characterized, catalytically inactive variant PPK-H435A (25). As shown in Fig. 1B, the expression of wild-type PPK fully rescued the cisplatin sensitivity of the *ppk* deletion strain. The expression of the catalytically inactive PPK variant, on the other hand, failed to rescue the growth defect. These results strongly suggested that endogenous levels of polyP are necessary and sufficient to protect E. coli against chronic cisplatin stress.

### PolyP protects E. coli against cisplatin-induced DNA damage.

To begin to understand how polyP protects bacteria against cisplatin toxicity, we analyzed the expression of select heat shock and SOS response genes in cisplatin-treated wild-type and *ppk* and *ppx* deletion mutant strains. This line of experiments was instigated by our previous study, which showed that during severe oxidative stress, polyP-deficient bacteria upregulate their heat shock gene expression levels in an apparent attempt to compensate for the lack of polyP’s chaperone function (19). Analysis of the mRNA levels of IbpA and DnaK, two genes whose expression levels are highly responsive toward protein unfolding stress in E. coli (26), did not reveal any significant change upon cisplatin treatment in any of the three tested strains (Fig. S1C). In contrast, however, we found that the mRNA levels of the gene encoding the cell division inhibitor SulA, a major component of the SOS response and an inducer of bacterial filamentation, significantly increased upon cisplatin treatment in the wild-type E. coli and *ppx* deletion strain and went up even more in bacteria lacking polyP. These results agreed with the original observation that cisplatin treatment triggers bacterial filamentation (27) and indicated that at the concentrations used, cisplatin works as a DNA rather than a protein-damaging reagent in bacteria.

To assess the levels of DNA damage that cisplatin elicits in the *ppk* deletion strain versus in bacteria that contain measurable levels of polyP, we determined the mutagenesis rates before and after cisplatin treatment by counting the number of bacteria that gain the ability to grow on rifampin plates (28). Rifampin-resistant mutations in the RNA polymerase gene *rpoB* arise from single base substitutions, which prevent rifampin from binding to and hence inhibiting RNA polymerase (29). Under nonstress conditions, the spontaneous rate of mutagenesis in all three tested strains was similarly low (<10 mutants per 10^8^ cells) (Fig. 1C). Not unexpectedly given cisplatin’s mode of action, this number drastically increased upon cisplatin treatment. However, the mutagenesis rate of the *ppk* deletion was almost 3-fold higher than the mutagenesis rates of cisplatin-treated wild-type E. coli or the *ppx* deletion strain. Expression of a highly active PPK variant, PPK-D230N, which substantially increases the steady-state levels of polyP *in vivo* (23), reduced the mutagenesis rate of the *ppk* deletion strain to levels that were even lower than the ones observed in cisplatin-treated wild-type E. coli (Fig. 1C). These results suggested a previously unrecognized function of polyP in bacterial DNA damage control.

### Cisplatin triggers extensive transcriptional changes in wild-type and polyP-depleted E. coli.

**FIG 2:**
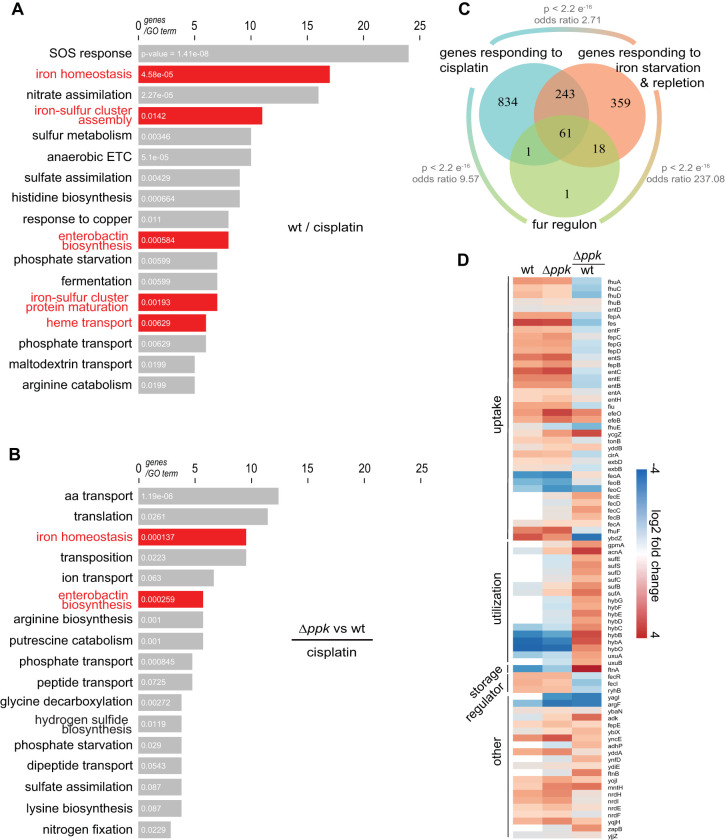
Global gene expression changes in response to cisplatin treatment. (A) Functional classification (GOterm) of genes differentially expressed in wild-type E. coli upon 15-min treatment with a nonlethal cisplatin concentration (20 μg/ml) (Tables S1 and S4). Categories related to iron metabolism are highlighted in red. (B) Functional classification (GOterm) of genes differentially expressed in *Δppk* and wild-type E. coli upon 15-min treatment with a nonlethal cisplatin concentration (20 μg/ml) (Tables S3 and S4). *P* values are shown in white on each bar, from a modified Fisher exact test. (C) Venn diagram of genes belonging to the *fur* regulon (green circle), genes differentially expressed in wild-type E. coli upon cisplatin treatment (cyan circle), and genes differentially expressed in a *fur* deletion strain under iron repletion and starvation conditions (22) (red circle). *P* values and odds ratios from Fisher exact tests. (D) Heatmap of Fur-regulated genes, which are differentially expressed in cisplatin-treated wild-type (wt) and *Δppk* cells. Data are log_2_ fold change, range −4 (blue) to 4 (red). Ratio of *ppk* to wild-type cells is shown in the right column. Genes are organized according to their functional annotations.

We conducted transcriptome sequencing (RNA-seq) analysis to compare the transcriptional profile of wild-type and *Δppk* cells in response to a sublethal dose of cisplatin (i.e., 20 μg/ml cisplatin for 15 min). Compared to the respective untreated controls, we identified more than 1,100 differentially expressed genes (DEGs) in each strain (see Tables S1 to S4). We categorized the DEGs into clusters of ontology according to their functional annotation (GOterm) (30) and focused our primary analysis on the two following groups: (i) DEGs in wild-type E. coli upon cisplatin treatment (Fig. 2A; Tables S1 and S4), since we reasoned that those genes will likely reveal the cellular effects that cisplatin exerts in bacteria; and (ii) DEGs in cisplatin-treated wild type versus Δ*ppk* strain (Fig. 2B; Tables S3 and S4), since we deduced that those genes will likely provide mechanistic insights into how polyP protects bacteria against cisplatin stress. The most upregulated genes in cisplatin-treated wild-type E. coli compared to the untreated control belonged to members of the SOS response (Fig. 2A and Table S1). This was not an unexpected result since the SOS response is the canonical response to DNA damage. It controls more than 40 genes involved in transcriptional regulation, DNA repair mechanism, cell cycle arrest, and error-prone DNA synthesis (31–33) (Fig. 2A). Many of the next most significantly altered cluster of DEGs in cisplatin-treated wild-type E. coli included genes involved in iron-sulfur cluster assembly, iron uptake, enterobactin synthesis, and iron regulation (Fig. 2A, red bars). These results suggested that cisplatin treatment causes a disturbance in the cellular iron homeostasis. Indeed, when we compared the DEGs in cisplatin-treated wild type with the DEGs identified under iron starvation or repletion conditions (22), we observed a statistically highly significant overlap (Fig. 2C). Particularly striking was the overlap between genes differentially regulated by cisplatin and genes previously reported to be directly controlled by the master transcriptional repressor *f*erric *u*ptake *r*egulator (FUR) (22) (Fig. 2C). In fact, the expression pattern in cisplatin-treated wild-type bacteria strongly resembled the expression pattern in E. coli mutants lacking functional FUR, i.e., the upregulation of genes involved in iron uptake and the selective downregulation of genes involved in iron utilization (Fig. 2D). These results suggested that cisplatin treatment affects iron homeostasis in wild-type bacteria, either by triggering or by signaling an iron starvation response. Other significantly enriched gene clusters in cisplatin-treated wild-type bacteria included genes involved in fermentation and anaerobic electron transport chain (ETC), as well as nitrate assimilation and sulfur and phosphate metabolism (Fig. 2A).

10.1128/mBio.01017-20.6 TABLE S1 RNA-seq analysis of wild-type E. coli MG1655 before and after 15-min cisplatin treatment. Download Table S1, XLSX file, 0.5 MB. Copyright © 2020 Beaufay et al. 2020 Beaufay et al. This content is distributed under the terms of the Creative Commons Attribution 4.0 International license.

10.1128/mBio.01017-20.7 TABLE S2 RNA-seq analysis of MG1655 *Δppk* before and after 15-min cisplatin treatment. Download Table S2, XLSX file, 0.5 MB. Copyright © 2020 Beaufay et al. 2020 Beaufay et al. This content is distributed under the terms of the Creative Commons Attribution 4.0 International license.

10.1128/mBio.01017-20.8 TABLE S3 List of differentially expressed genes in cisplatin-treated wild-type E. coli MG1655 and MG1655 *Δppk*. Download Table S3, XLSX file, 0.4 MB. Copyright © 2020 Beaufay et al. 2020 Beaufay et al. This content is distributed under the terms of the Creative Commons Attribution 4.0 International license.

10.1128/mBio.01017-20.9 TABLE S4 GOterm analysis of DEGs in wild-type E. coli MG1655 and MG1655 *Δppk* upon cisplatin treatment. Download Table S4, XLSX file, 0.02 MB. Copyright © 2020 Beaufay et al. 2020 Beaufay et al. This content is distributed under the terms of the Creative Commons Attribution 4.0 International license.

### Cisplatin elicits iron stress in polyP-deficient E. coli.

**FIG 3:**
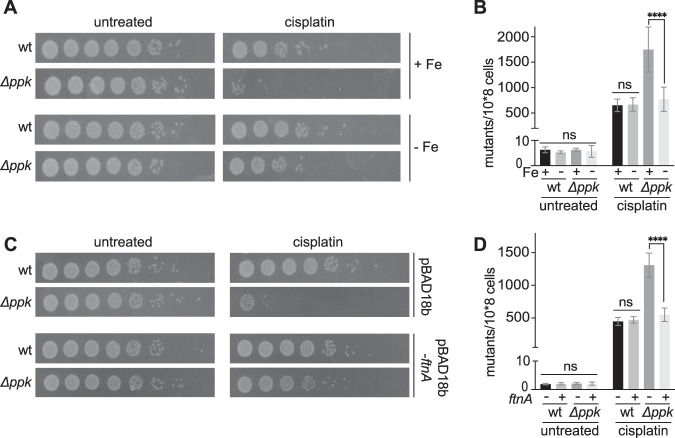
Low-iron conditions reduce cisplatin toxicity in polyP-deficient E. coli. (A and B) Exponentially growing E. coli wild-type (wt) and *ppk* deletion strains were exposed to 10 μg/ml cisplatin in untreated (+Fe) or Chelex treated (−Fe) M9-G medium for 20 h to determine survival (A) or for 1 h to determine the rate of mutagenesis (B). (C and D) Exponentially growing E. coli wild-type and *ppk* deletion strains carrying either an empty plasmid or a plasmid overexpressing the iron storage protein FtnA were exposed to 10 μg/ml cisplatin in M9-G medium for either 20 h to determine survival (C) or 1 h to determine the rate of mutagenesis (D).

Direct comparison of the DEGs in cisplatin-treated wild-type E. coli and polyP-deficient mutant cells revealed numerous gene clusters that responded similarly to cisplatin treatment in the two strains (compare Fig. 2A with Fig. S2A; Table S3). In stark contrast, however, we found dramatic differences between the two strains in the expression of genes involved in amino acid transport, translation, transposition, and, particularly, iron homeostasis (Fig. 2B). Given the close connection between iron, oxidative stress, and DNA damage, we subsequently focused on the DEGs associated with iron homeostasis. We observed that polyP*-*depleted cells respond to cisplatin treatment with a much less pronounced expression of iron uptake genes and an even more pronounced downregulation of iron utilization genes (Fig. 2D and Table S3). This expression pattern suggested that polyP-depleted bacteria experience a relative increase in the intracellular labile iron pool upon cisplatin treatment, which would explain their relative decrease in survival and increase in mutagenesis rate compared to wild-type bacteria (Fig. 1A and C). Given the polyanionic structure of polyP, and its previously shown ability to interact with divalent metals, such as Ca^2+^, Mg^2+^, and certain heavy metals (34, 35), we therefore considered the possibility that polyP serves as a hitherto unknown iron chelator. PolyP might complex excess iron as it is being taken up from the extracellular space and/or released from iron-containing proteins during cisplatin treatment. We reasoned that if this model were to be correct, we should be able to specifically rescue the cisplatin-sensitivity of the *ppk* deletion strain by reducing the intracellular iron load during cisplatin treatment. To test this idea, we devised two different strategies: (i) decreasing the extracellular iron concentration, which should reduce the amount of Fe uptake during cisplatin stress (36), or (ii) overexpressing the iron storage protein FtnA, which should compensate for the lack of polyP. For our first strategy, we grew wild-type and *ppk* deletion strains in liquid medium supplemented with either normal (+Fe) or low (−Fe) concentrations, exposed them to our previously established cisplatin treatment, and determined cell survival and mutagenesis rates. The result that we obtained was fully consistent with our hypothesis: lowering the iron concentration in the media dramatically increased the survival and reduced the mutagenesis rates of the *ppk* deletion strain to levels directly comparable to cisplatin-treated E. coli wild type (Fig. 3A and B). In contrast, cisplatin treatment under low versus normal iron conditions did not yield any noticeable difference in the cisplatin resistance of wild-type E. coli. These results suggested that iron taken up from the media in response to cisplatin treatment was successfully complexed or otherwise neutralized by polyP. We obtained a very similar result upon overexpression of the plasmid-encoded iron storage protein FtnA, which significantly improved survival and reduced the mutagenesis rates in the cisplatin-treated *ppk* deletion strain but not in wild-type E. coli (Fig. 3C and D). These results demonstrated that expression of a protein-based iron chelator fully compensates for the lack of polyP and suggested that iron complexed in metalloproteins might contribute to the iron toxicity in the absence of polyP. Since previous reports documented that cisplatin covalently binds cysteine residues in proteins (9, 13, 37), including those involved in iron binding (38), we finally tested the idea that cisplatin targets iron-containing proteins in bacterial cell lysates. We therefore prepared wild-type or *Δppk* crude extracts and measured the activity of aconitase, an enzyme, whose iron-sulfur cluster is coordinated via three oxidation-sensitive cysteines. We found that increasing concentrations of cisplatin indeed increasingly reduced the activity of aconitase at similar levels in both wild-type and *Δppk* extracts (Fig. S3). Together, these results suggested that endogenous polyP provides an effective nonproteinogenic mechanism to protect bacteria against cisplatin-mediated accumulation of free iron and Fe-mediated oxidative damage.

10.1128/mBio.01017-20.3 FIG S2 Gene enrichment analysis in *Δppk* E. coli in response to cisplatin. Functional classification (GOterm) of genes differentially expressed upon cisplatin treatment in E. coli *Δppk*. Categories related to iron metabolism are highlighted in red. *P* values are shown in white over each bar, using a modified Fisher exact test (see Table S2 for input). Download FIG S2, EPS file, 1.3 MB. Copyright © 2020 Beaufay et al. 2020 Beaufay et al. This content is distributed under the terms of the Creative Commons Attribution 4.0 International license.

10.1128/mBio.01017-20.4 FIG S3 Cisplatin inhibits the iron-sulfur cluster protein aconitase. (A) Influence of cisplatin on the aconitase activity in crude extracts from either wild-type or *Δppk* cells. The cell lysates were exposed to either 0, 10, 20, or 50 μg/ml cisplatin at 37°C. After 1 h of treatment, the remaining aconitase activity was determined. The aconitase activity in untreated lysates was set to 100%. The mean ± standard deviation from 3 measurements is shown. Download FIG S3, EPS file, 1.1 MB. Copyright © 2020 Beaufay et al. 2020 Beaufay et al. This content is distributed under the terms of the Creative Commons Attribution 4.0 International license.

### PolyP acts as a physiologically relevant iron storage molecule *in vivo*.

**FIG 4:**
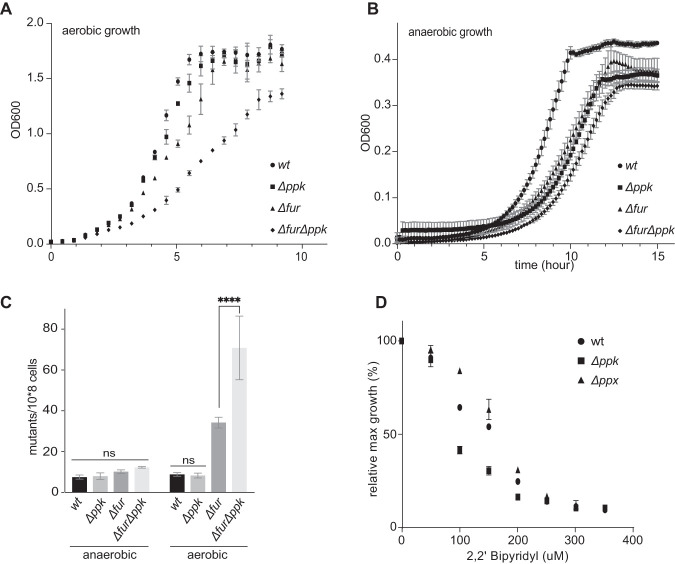
PolyP acts as an iron-storage molecule *in vivo.* (A and B) Growth of E. coli wild-type, *Δppk*, *Δfur*, and *Δfur Δppk* strains in MOPS-G medium under aerobic (A) or anaerobic (B) conditions. (C) Mutagenesis rate of each strain under aerobic and anaerobic growth conditions. (*n* = 3; ****, *P* < 0.0001; ns, nonsignificant; one-way ANOVA). (D) Exponentially growing E. coli wild-type and *Δppk* and *Δppx* deletion strains were diluted into M9-gluconate supplemented with increasing concentrations of the iron chelator 2,2′-bipyridyl. The maximal growth achieved after 18 h of incubation at 37°C was normalized against the growth in M9-gluconate without chelator. Error bars are ±1 standard deviation for technical triplicates. Experiments were performed in triplicates (for biological replicates, see Fig. S4A and B), and a representative result is shown.

Iron homeostasis is a tightly regulated mechanism, necessitated by the fact that unbound labile iron is highly toxic under aerobic growth conditions. This toxicity appears to be primarily caused by the ability of free iron to interact with peroxide (i.e., Fenton reaction), which leads to the production of highly reactive hydroxyl radicals (39). To further evaluate the idea that polyP serves as a general, hitherto unrecognized iron storage molecule in bacteria, we turned to a mutant strain of E. coli, which lacks the master iron repressor Fur. Deletion of Fur triggers an iron starvation response, which, similar to the situation we observed in cisplatin-treated wild-type E. coli, leads to gene expression changes that are geared toward replenishing the intracellular iron pool (40). As a cellular consequence, *fur* deletion strains suffer from an intracellular iron overload, which causes severe growth defects and significantly higher mutagenesis rates under aerobic but not under anaerobic growth conditions (28). To test whether polyP functions as an iron storage molecule in a *Δfur* deletion strain, we generated *Δfur Δppk* double deletion strains and analyzed growth and mutagenesis rates under both aerobic and anaerobic growth conditions. As shown in Fig. 4A to C, additional deletion of *ppk* in the *Δfur* deletion strain significantly aggravated the growth defect and increased the mutagenesis rate specifically under aerobic growth conditions (Fig. 4A to C). These results were fully consistent with our prior observations and supported our conclusion that polyP protects bacteria generally against conditions of Fe overload. To finally test whether polyP also serves as an iron reservoir under nonstress conditions, we cultivated E. coli wild-type, *Δppk*, and *Δppx* strains in M9 minimal medium in the presence of increasing concentrations of the divalent iron chelator 2,2′-dipyridyl, using gluconate as sole carbon source. By offering solely gluconate, we ensure that bacterial growth depends on the activity of the iron-sulfur cluster protein gluconate dehydratase and hence on the availability of intracellular iron (41). Addition of 2,2′-dipyridyl to the growth media reduces the intracellular iron pools and, at sufficiently high levels, prevents bacterial growth as it depletes the Fe-S cluster in gluconate dehydratase. As shown in Fig. 4D (and Fig. S4A and B), whereas the *Δppx* strain was slightly more resistant toward the presence of the chelator compared to wild-type E. coli, the *Δppk* strain was significantly more sensitive. Less than 100 μM 2,2′-dipyridyl in the growth medium was sufficient to decrease the relative growth rate of the *Δppk* strain by 50% while more than 150 μM chelator was necessary to trigger the same growth defect in wild-type E. coli (Fig. 4D and Fig. S4A and B). These results demonstrated that lack of endogenous polyP drastically increases the sensitivity of E. coli toward the presence of iron chelators in the media and supports the conclusion that polyP serves as a physiologically relevant iron storage molecule in E. coli.

10.1128/mBio.01017-20.5 FIG S4 Biological replicates of data shown in Fig. 4D. (A and B) Exponentially growing E. coli wild-type, *ppk*, and *ppx* deletion strains were diluted into M9-gluconate medium supplemented with increasing concentrations of the iron chelator 2,2′-dipyridyl. The maximal growth after an 18-h incubation at 37°C was normalized against the growth in M9-gluconate without chelator. Error bars are ±1 standard deviation for technical triplicates. Download FIG S4, EPS file, 1.2 MB. Copyright © 2020 Beaufay et al. 2020 Beaufay et al. This content is distributed under the terms of the Creative Commons Attribution 4.0 International license.

### PolyP protects against Fe-mediated DNA damage *in vitro*.

**FIG 5:**
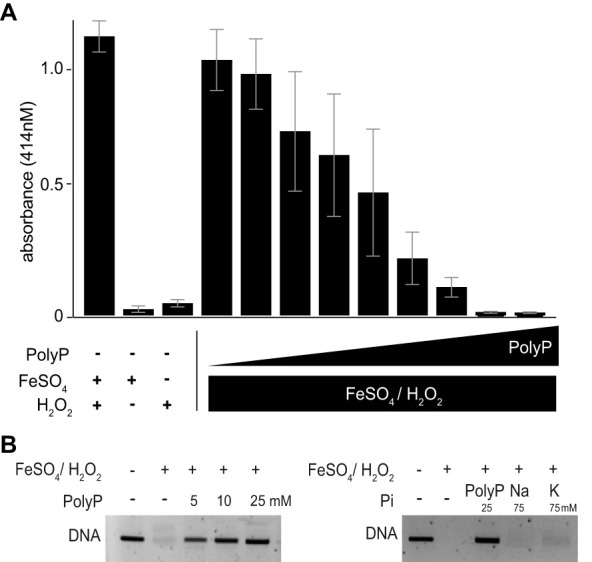
PolyP inhibits Fenton reaction *in vitro.* (A) ABTS oxidation by 20 μM H_2_O_2_ and 5 μM FeSO_4_ was monitored at 414 nm in the absence or presence of 1, 2, 3, 4, 5, 7.5, 10, 20, or 30 μM polyP_300_ (in P_i_ units). No significant oxidation was observed in the presence of H_2_O_2_ or FeSO_4_ alone (*n* = 3). (B) Oxidative degradation of 200 ng of DNA after 30-min incubation with 2 mM H_2_O_2_ and 50 μM FeSO_4_ in the absence or presence of 5, 10, or 25 mM polyP_300_ (in P_i_ units); 75 mM NaH_2_PO_4_; or 75 mM KH_2_PO_4_. Samples were applied onto an agarose gel and visualized using ethidium bromide (*n* = 3).

To directly test whether polyP, through complexing iron, mitigates the Fenton reaction, we monitored the H_2_O_2_/FeSO_4_-mediated oxidation of 2,2′-azino-bis(3-ethylbenzothiazoline-6-sulfonic acid) (ABTS) (42) in the presence of increasing amounts of polyP (Fig. 5A). ABTS, once oxidized by H_2_O_2_/FeSO_4_-produced hydroxyl radicals, shows a strong absorbance signal at 414 nm. As shown in Fig. 5A, the presence of polyP prevented ABTS oxidation in a concentration-dependent manner, indicating that chelation of Fe^2+^ by polyP inhibits the Fenton reaction. In fact, 20 μM polyP_300_ (in P_i_ units) was sufficient to prevent oxidation of ABTS by a mixture of 5 μM FeSO_4_ and 20 μM H_2_O_2_ (Fig. 5A). To test whether the association of Fe^2+^ with polyP also protects DNA against Fe-mediated oxidative damage, we incubated 10 μM linearized DNA with a mixture of 50 μM Fe^2+^/5 mM H_2_O_2_ in the absence and presence of polyP_300_ (Fig. 5B). Whereas in the absence of polyP, all of the DNA was oxidatively degraded within a 30-min incubation period, the presence of 5 mM polyP_300_ (in P_i_ units) almost completely prevented the degradation of DNA. The presence of inorganic phosphate (P_i_) in the form of either sodium phosphate or potassium phosphate did not have any protective effect even when used at concentrations as high as 75 mM (Fig. 5B), confirming that the polyanionic nature of the polyP chain is necessary to sequester iron in a nonreactive state. In summary, these results demonstrate that polyP acts as a physiologically relevant iron storage molecule, capable of preventing the production of hydroxyl radical by the Fenton reaction.

## DISCUSSION

**FIG 6:**
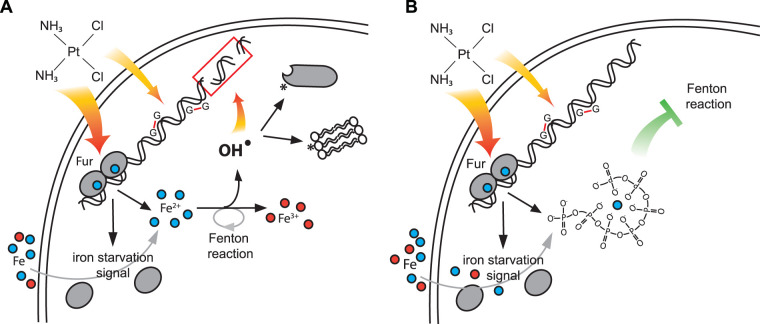
Model for the protective effects of polyphosphate against cisplatin. (A) Our data suggest that cisplatin treatment causes iron accumulation that might contribute to a second mode of killing through Fenton-mediated oxidative stress and DNA damage. Iron stress likely results from a combination of events: the release of protein-bound iron and the inactivation of the master repressor of bacterial iron homeostasis Fur that triggers gene expression changes aimed to increase the cellular iron load. Subsequently, cellular free iron levels increase and can wreak havoc under aerobic growth conditions by producing highly reactive oxygen species, such as hydroxyl radicals. (B) Due to its polyanionic nature, endogenous polyP binds free iron and prevents iron from acting as a catalyst for the Fenton reaction, thereby drastically reducing the toxicity of cisplatin.

Iron serves as an essential cofactor in many enzymes, which are involved in processes ranging from metabolism to DNA synthesis and cell division. Yet, free iron is highly toxic under oxygen-rich conditions as it readily undergoes the Fenton or iron-catalyzed Haber-Weiss reaction, thereby producing extremely reactive oxygen species, particularly hydroxyl radicals (39) (Fig. 6A). Because of this dichotomy in cellular need for and risk of free iron, aerobically growing organisms such as E. coli tightly regulate their iron homeostasis. Although the transcriptional regulation of iron homeostasis has been well studied over the years (43), little is known about potential first responders, such as physiological iron chelators, that could buffer the cellular effects of iron deregulation. Here, we demonstrate that cisplatin, an anticancer drug and broad-spectrum antimicrobial, triggers cytotoxicity in bacteria not only through its ability to cross-link DNA but also by causing cellular iron overload. By using a combination of genetic and biochemical tools, we discovered that bacteria defend themselves against this secondary insult through polyP, which effectively complexes the iron, prevents the Fenton reaction, and mitigates cisplatin toxicity (Fig. 6B).

It is well established that polyP interacts with metals such as Ca^2+^ to form acidocalcisomes and protects organisms by complexing and sequestrating heavy metals (44–46). In contrast, however, very little is known about the interaction of polyP with iron, the physiological relevance of such an interaction, or its potential role in the Fenton reaction (47, 48). In fact, earlier *in vitro* studies suggested that polyP as well as shorter phosphate moieties, including pyro-, tri-, and tetrapolyphosphate, might actually stimulate the Fenton reaction (49, 50). This result agreed with detailed follow-up studies, aimed to identify physiologically relevant Fenton inhibitors. This study revealed that nucleotide phosphates, such as ATP, either stimulate or prevent the Fenton reaction, depending on the number of iron coordination sites that were occupied by phosphates in the complex (48). The most effective Fenton reaction inhibitor *in vitro* turned out to be dimers of ATP-y-S and inositol-6-phosphate, which, through their six existing phosphate groups, block all iron coordination sites (48, 51). This appears to prevent H_2_O_2_ from reacting with iron and mitigates hydroxyl radical formation. We now propose that polyP, due to its polyanionic nature and structural flexibility, is able to also occupy all relevant coordination sites in iron, thereby interfering with peroxide binding and preventing the Fenton reaction (Fig. 6B). This feature would make polyP, to our knowledge, one of the few known physiologically relevant, nonproteinaceous, Fenton reaction inhibitors in living cells.

Our studies revealed noteworthy parallels between the protective function of polyP in bacteria exposed to cisplatin and in bacteria that lack the iron repressor Fur. Our transcriptional analysis of cisplatin-treated wild-type bacteria supported this conclusion by demonstrating that close to 80% of previously identified Fur regulons are upregulated in bacteria treated with cisplatin (22). At first glance, these results suggested that bacteria treated with cisplatin suffer from stress conditions that trigger intracellular iron depletion; hence, the upregulation of siderophores and transport mechanisms aimed to replenish the intracellular iron pools. However, analysis of the cisplatin response in polyP-depleted bacteria yielded quite the opposite result and, in fact, suggested that cisplatin triggers an accumulation of free iron, which becomes highly toxic and mutagenic unless chelated by polyP. Further support for this conclusion came from cisplatin treatment studies in Fe-depleted media, as well as complementation studies in which we overexpressed the E. coli Fe storage protein FtnA. In both scenarios, cisplatin treatment of the *ppk* deletion strain no longer increased the mutagenesis rate or affected survival beyond what we observed in cisplatin-treated wild-type E. coli. These results strongly argue that cisplatin treatment causes an increase in labile intracellular iron, which, unless chelated by polyP, significantly increases the toxicity of cisplatin. Since cisplatin is well known for its ability to interact with and bind to cysteine and methionine residues in proteins (11), and Fur contains a redox-sensitive cysteine-coordinating zinc site (52), we now speculate that Fur itself might become a target of cisplatin. Inactivation of Fur would misleadingly send an iron starvation signal to the cell, causing iron accumulation. Intriguingly, very recent studies showed similarly disruptive effects of cisplatin on the iron homeostasis in cancer cells (53). In contrast to bacteria, however, which ultimately suffer from iron overload, cisplatin-treated cancer cells experience true iron starvation. This iron starvation phenotype is triggered by the covalent modification of two cysteines in the iron regulatory protein 2 (IRP2), a central activator of the mammalian iron starvation response (53). Once inhibited, IRP2 is unable to downregulate the iron chelator ferritin, causing persistent iron depletion. Our realization that polyP serves as an iron chelator helps to explain our recent finding that endogenous levels of polyP positively correlate with apoptosis in cisplatin-treated cancer cells (16). We now reason that by chelating iron, polyP further potentiates the iron starvation phenotype in cancer cells, hence causing the observed increase in cytotoxicity (16, 53).

The ability of polyP to complex iron might also contribute to its protective function under oxidative stress conditions, as well as other cellular insults that cause protein-bound iron to be released. Relying on nonproteinaceous compounds to chelate a toxic but essential metal might reflect a global strategy of the stress response. PolyP accumulation does not require time-consuming transcription or translation processes, and the molecule itself is stable toward almost all relevant damaging agents (18). Moreover, polyP ensures the proper activation of RpoS, the master regulator of the general stress response in E. coli leading to the expression of genes such as catalases to increase the overall resistance to oxidative stress (54). In addition, chelation of iron by polyP would allow iron to be rapidly reincorporated into proteins without the activation of complex and energy-consuming uptake systems once the stress is removed. It now remains to be tested, however, whether and how association of polyP with iron affects some of the other known functions of polyP, such as its ability to interact with and stabilize unfolding proteins, or its activity in blood coagulation and inflammation (55). This would add another exciting new layer of complexity to this structurally simple molecule.

## MATERIALS AND METHODS

### Bacterial strains and growth conditions.

All strains, plasmids, and oligonucleotides used in this study are listed in Table S5 in the supplemental material. Gene deletions were generated by λ red-mediated site-specific recombination (56). All chromosomal mutations were confirmed by PCR. E. coli MG1655 was grown at 37°C in lysogenic broth (LB; Fisher) or MOPS minimal medium (Teknova) containing 0.2% (wt/vol) glucose and 1.32 mM K_2_HPO_4_ (MOPS-G). To conduct cisplatin resistance tests on plates, M9 minimal medium (57) containing 0.2% glucose (M9-G) was supplemented with 1.5% agar. When indicated, 0.2% (wt/vol) gluconate was used instead of glucose (M9-gluconate). For iron-depleted conditions, M9-G medium was mixed and incubated with 2 g/liter Chelex100 (Bio-Rad) for 1 h at room temperature under constant agitation. The chelated solution was then sterile filtered. For pBAD expression induction, arabinose (0.2%, wt/vol) was added 15 min prior to the experiment in the case of liquid assays or added to the media in the case of plate assays. The following antibiotics were added when appropriate: chloramphenicol (30 μg/ml), rifampin (200 μg/ml), kanamycin (50 μg/ml), or ampicillin (100 μg/ml).

10.1128/mBio.01017-20.10 TABLE S5 Strains, plasmids, and oligonucleotides used in this study. Download Table S5, DOCX file, 0.02 MB. Copyright © 2020 Beaufay et al. 2020 Beaufay et al. This content is distributed under the terms of the Creative Commons Attribution 4.0 International license.

### Growth under iron starvation.

Iron starvation growth assays were performed as described in reference 58. Briefly, E. coli MG1655 wild-type and *ppk* and *ppx* deletion strains were grown overnight in M9-gluconate medium, diluted, and cultivated at 37°C until an OD_600_ of 0.5 was reached. Cells were then diluted 1:100 into M9-gluconate medium supplemented with the indicated concentrations of 2,2′-dipyridyl (stock dissolved in 100 mM dimethyl sulfoxide [DMSO]; Sigma-Aldrich). Each condition was performed in triplicate, and growth was monitored for 18 h. The OD_600_ reached after 18 h was then normalized to the corresponding growth rate in M9-gluconate in the absence of chelator and plotted.

### Cisplatin survival assay.

E. coli MG1655 wild type and the isogenic mutant strains were grown at 37°C with shaking in MOPS-G medium to an OD_600_ of ∼0.4 to 0.8 and harvested by centrifugation. To analyze cisplatin sensitivity in liquid culture, the cells were resuspended in MOPS-G medium to an OD_600_ of 0.4, supplemented with various concentrations of cisplatin (stock solution 0.9 mg/ml in sterile double-distilled water [ddH_2_O]; Sigma-Aldrich), and incubated at 37°C with shaking (200 rpm). At defined time points of incubation (1 h to 20 h), the cells were harvested by centrifugation, washed twice, 10-fold serially diluted, and plated onto LB agar. Survival was assessed after overnight incubation at 37°C. To determine the survival of bacteria when grown on cisplatin-containing plates, bacteria were cultivated in MOPS-G medium until an OD_600_ of 0.5 was reached. Then, the bacteria were 10-fold serially diluted and plated onto M9-G plates containing various concentrations of cisplatin. Colonies were counted after 24 h of incubation at 37°C. To compare the cisplatin sensitivities of bacteria in iron-depleted liquid media, the bacteria were first grown in MOPS-G medium as described above. Once an OD_600_ of 0.4 was reached, the bacteria were centrifuged, washed, and resuspended in either untreated M9-G (+Fe) medium or Chelex-treated M9-G (−Fe) medium in the absence or presence of 10 μg/ml of cisplatin. At defined time points of incubation (1 h to 20 h), the cells were harvested by centrifugation, washed twice, 10-fold serially diluted, and plated onto LB agar.

### Mutagenesis assay.

Mutagenesis rates were measured as described previously (59). Briefly, cells were grown overnight in MOPS-G medium, diluted 1:100 into 30 ml fresh MOPS-G medium, and cultivated at 37°C until an OD_600_ of 0.5 was reached. The bacterial suspension was either left untreated or supplemented with 10 μg/ml cisplatin and further incubated for 1 h. After the incubation, untreated and treated bacteria were washed twice in MOPS-G medium and resuspended in 5 ml of LB medium. The bacteria were then incubated overnight at 37°C with shaking. Serial dilutions were made and plated onto both LB agar and LB-rifampin agar plates. After 24 h of incubation at 37°C, the CFU were scored. The mutation frequency was calculated by dividing the number of rifampin-positive colonies by the total number of colonies.

### RNA-seq analysis.

Four biological replicates of wild-type E. coli MG1655 and *Δppk* mutant were cultivated in MOPS-G medium at 37°C until an OD_600_ of 0.4 to 0.5 was reached. Cells (1 ml) were harvested either before or 15 min after the treatment with 20 μg/ml cisplatin in 1 ml of ice-cold methanol (−80°C) to stop transcription. After centrifugation and removal of the supernatant, total RNA was prepared using the Ambion RiboPure-Bacteria kit (Thermo Fisher Scientific) according to the manufacturer’s instructions. The samples were DNase I treated, followed by depletion of rRNA using the Illumina Ribo Zero kit (Illumina) for Gram-negative bacteria. Fifty-base single-end sequencing was performed on an Illumina HiSeq 4000 using the University of Michigan DNA Sequencing Core. Sequence reads from the RNA-seq were mapped onto the reference genome (NC_000913). Genes with a log_2_ fold change of >0.5 and a false-discovery rate (FDR) value of <0.01 were considered differentially expressed genes (DEGs).

### COG enrichment and analysis.

Differentially regulated genes upon cisplatin treatment and/or between strains were categorized according to their annotated COG categories (30). Functional enrichment of COG categories was determined by performing a modified one-tailed Fisher exact test (EASE score from DAVID), with a *P* value of <0.05 considered significant. Comparison to iron-regulated genes (22) seen in the Venn diagram in Fig. 2C was performed using a Fisher exact test. The heatmap was produced using open-source statistical software R (https://www.r-project.org/) with log_2_ fold change data. Figures 2 and S2 were also produced in R.

### Enzymatic assays.

The aconitase activity assay was performed according to the manufacturer’s protocol (MAK051; Sigma-Aldrich). Briefly, bacterial cell pellets were resuspended in 1 ml of aconitase lysis buffer (0.1 mM Tris-HCl, pH 8.0, 0.1 M KCl, 1 mM phenylmethylsulfonyl fluoride [PMSF], and 0.6 μg/μl lysozyme) and lysed by 5 to 6 rounds of freeze-and-thaw cycles. The lysates were centrifuged at 14,000 rpm for 10 min at 4°C. The total protein concentration was determined using the Bradford assay. The aconitase assay was performed by mixing 100 μg of cell lysate with 200 μl of 1× aconitase assay buffer (0.6 mM MnCl_2_, 25 mM sodium citrate, 0.25 mm ADP, 50 mM Tris-HCl, pH 7.6). The absorbance was recorded at 340 nm, and the specific aconitase activity was calculated per milligram of total proteins.

### *In vitro* Fenton reaction and DNA damage assay.

The ABTS assay was performed as previously described in reference 42. In brief, 250 μM ABTS was mixed with 5 μM FeSO_4_ and 200 μM H_2_O_2_ in the absence or presence of increasing concentrations of polyP_300_ (see figure legends for details). The assay was performed in 10 mM acetate buffer, pH 3.6, to control acidity. After 30 min of incubation at 37°C, the mixture was read at an absorbance of 414 nm. The DNA damage assay was performed by mixing 50 μM FeSO_4_, 5 mM H_2_O_2_, and 10 μM linearized DNA from the plasmid pBAD18 in water with the indicated concentration of either Na_2_HPO_4_, KH_2_PO_4_, or polyP_300_ (concentration given in P_i_ units). The reaction was initiated by the addition of H_2_O_2_, and the reaction mixture was incubated for 30 min at 37°C. Then, samples were loaded onto an agarose gel. Staining with ethidium bromide was used to visualize the DNA bands.

10.1128/mBio.01017-20.1 TEXT S1 Supplemental materials and methods. Download Text S1, DOCX file, 0.02 MB. Copyright © 2020 Beaufay et al. 2020 Beaufay et al. This content is distributed under the terms of the Creative Commons Attribution 4.0 International license.

## References

[1] Galanski M, Slaby S, Jakupec MA, Keppler BK 2005 Synthesis and in vitro antitumor potency of (cyclohexane-1,2-diamine)platinum(II) complexes with aminotris(methylenephosphonic acid) as bone-seeking ligand. Bioinorg Chem Appl 3:179–190. doi:10.1155/BCA.2005.179. PMC2267107 18365098

[2] Hannon MJ 2007 Metal-based anticancer drugs: from a past anchored in platinum chemistry to a post-genomic future of diverse chemistry and biology. Pure Appl Chem 79:2243–2261. doi:10.1351/pac200779122243.

[3] Eastman A 1987 The formation, isolation and characterization of DNA adducts produced by anticancer platinum complexes. Pharmacol Ther 34:155–166. doi:10.1016/0163-7258(87)90009-x. 3317449

[4] Bancroft DP, Lepre CA, Lippard SJ 1990 Platinum-195 NMR kinetic and mechanistic studies of cis-and trans-diamminedichloroplatinum (II) binding to DNA. J Am Chem Soc 112:6860–6871. doi:10.1021/ja00175a020.

[5] Sherman SE, Lippard SJ 1987 Structural aspects of platinum anticancer drug interactions with DNA. Chem Rev 87:1153–1181. doi:10.1021/cr00081a013.

[6] Mandic A, Hansson J, Linder S, Shoshan MC 2003 Cisplatin induces endoplasmic reticulum stress and nucleus-independent apoptotic signaling. J Biol Chem 278:9100–9106. doi:10.1074/jbc.M210284200. 12509415

[7] Yang Z, Schumaker LM, Egorin MJ, Zuhowski EG, Guo Z, Cullen KJ 2006 Cisplatin preferentially binds mitochondrial DNA and voltage-dependent anion channel protein in the mitochondrial membrane of head and neck squamous cell carcinoma: possible role in apoptosis. Clin Cancer Res 12:5817–5825. doi:10.1158/1078-0432.CCR-06-1037. 17020989

[8] Lin X, Okuda T, Holzer A, Howell SB 2002 The copper transporter CTR1 regulates cisplatin uptake in Saccharomyces cerevisiae. Mol Pharmacol 62:1154–1159. doi:10.1124/mol.62.5.1154. 12391279

[9] Ishikawa T, Ali-Osman F 1993 Glutathione-associated cis-diamminedichloroplatinum (II) metabolism and ATP-dependent efflux from leukemia cells. Molecular characterization of glutathione-platinum complex and its biological significance. J Biol Chem 268:20116–20125. 8376370

[10] Karasawa T, Sibrian-Vazquez M, Strongin RM, Steyger PS 2013 Identification of cisplatin-binding proteins using agarose conjugates of platinum compounds. PLoS One 8:e66220. doi:10.1371/journal.pone.0066220. 23755301 PMC3670892

[11] Peleg-Shulman T, Najajreh Y, Gibson D 2002 Interactions of cisplatin and transplatin with proteins: comparison of binding kinetics, binding sites and reactivity of the Pt-protein adducts of cisplatin and transplatin towards biological nucleophiles. J Inorg Biochem 91:306–311. doi:10.1016/s0162-0134(02)00362-8. 12121789

[12] Holford J, Beale P, Boxall F, Sharp S, Kelland L 2000 Mechanisms of drug resistance to the platinum complex ZD0473 in ovarian cancer cell lines. Eur J Cancer 36:1984–1990. doi:10.1016/s0959-8049(00)00192-1. 11000581

[13] Kelland L 2007 The resurgence of platinum-based cancer chemotherapy. Nat Rev Cancer 7:573–584. doi:10.1038/nrc2167. 17625587

[14] Kelley SL, Basu A, Teicher BA, Hacker MP, Hamer DH, Lazo JS 1988 Overexpression of metallothionein confers resistance to anticancer drugs. Science 241:1813–1815. doi:10.1126/science.3175622. 3175622

[15] Santos N, Catao C, Martins N, Curti C, Bianchi MLP, Santos ACD 2007 Cisplatin-induced nephrotoxicity is associated with oxidative stress, redox state unbalance, impairment of energetic metabolism and apoptosis in rat kidney mitochondria. Arch Toxicol 81:495–504. doi:10.1007/s00204-006-0173-2. 17216432

[16] Xie L, Rajpurkar A, Quarles E, Taube N, Rai AS, Erba J, Sliwinski B, Markowitz M, Jakob U, Knoefler D 2019 Accumulation of nucleolar inorganic polyphosphate is a cellular response to cisplatin-induced apoptosis. Front Oncol 9:1410. doi:10.3389/fonc.2019.01410. 31921667 PMC6920253

[17] Xie L, Jakob U 2019 Inorganic polyphosphate, a multifunctional polyanionic protein scaffold. J Biol Chem 294:2180–2190. doi:10.1074/jbc.REV118.002808. 30425096 PMC6369292

[18] Rao NN, Gómez-García MR, Kornberg A 2009 Inorganic polyphosphate: essential for growth and survival. Annu Rev Biochem 78:605–647. doi:10.1146/annurev.biochem.77.083007.093039. 19344251

[19] Gray MJ, Wholey W-Y, Wagner NO, Cremers CM, Mueller-Schickert A, Hock NT, Krieger AG, Smith EM, Bender RA, Bardwell JCA, Jakob U 2014 Polyphosphate is a primordial chaperone. Mol Cell 53:689–699. doi:10.1016/j.molcel.2014.01.012. 24560923 PMC3996911

[20] Cremers CM, Knoefler D, Gates S, Martin N, Dahl J-U, Lempart J, Xie L, Chapman MR, Galvan V, Southworth DR, Jakob U 2016 Polyphosphate: a conserved modifier of amyloidogenic processes. Mol Cell 63:768–780. doi:10.1016/j.molcel.2016.07.016. 27570072 PMC5234082

[21] Gray MJ, Jakob U 2015 Oxidative stress protection by polyphosphate—new roles for an old player. Curr Opin Microbiol 24:1–6. doi:10.1016/j.mib.2014.12.004. 25589044 PMC4380828

[22] Seo SW, Kim D, Latif H, O’Brien EJ, Szubin R, Palsson BO 2014 Deciphering Fur transcriptional regulatory network highlights its complex role beyond iron metabolism in Escherichia coli. Nat Commun 5:4910. doi:10.1038/ncomms5910. 25222563 PMC4167408

[23] Rudat AK, Pokhrel A, Green TJ, Gray MJ 2018 Mutations in Escherichia coli polyphosphate kinase that lead to dramatically increased in vivo polyphosphate levels. J Bacteriol 200:e00697-17. doi:10.1128/JB.00697-17. 29311274 PMC5826030

[24] Rao NN, Kornberg A 1996 Inorganic polyphosphate supports resistance and survival of stationary-phase Escherichia coli. J Bacteriol 178:1394–1400. doi:10.1128/jb.178.5.1394-1400.1996. 8631717 PMC177814

[25] Kumble KD, Ahn K, Kornberg A 1996 Phosphohistidyl active sites in polyphosphate kinase of Escherichia coli. Proc Natl Acad Sci U S A 93:14391–14395. doi:10.1073/pnas.93.25.14391. 8962061 PMC26142

[26] Guisbert E, Yura T, Rhodius VA, Gross CA 2008 Convergence of molecular, modeling, and systems approaches for an understanding of the Escherichia coli heat shock response. Microbiol Mol Biol Rev 72:545–554. doi:10.1128/MMBR.00007-08. 18772288 PMC2546862

[27] Rosenberg B, Vancamp L, Krigas T 1965 Inhibition of cell division in Escherichia coli by electrolysis products from a platinum electrode. Nature 205:698–699. doi:10.1038/205698a0. 14287410

[28] Touati D, Jacques M, Tardat B, Bouchard L, Despied S 1995 Lethal oxidative damage and mutagenesis are generated by iron in delta fur mutants of Escherichia coli: protective role of superoxide dismutase. J Bacteriol 177:2305–2314. doi:10.1128/jb.177.9.2305-2314.1995. 7730258 PMC176885

[29] Campbell EA, Korzheva N, Mustaev A, Murakami K, Nair S, Goldfarb A, Darst SA 2001 Structural mechanism for rifampicin inhibition of bacterial RNA polymerase. Cell 104:901–912. doi:10.1016/s0092-8674(01)00286-0. 11290327

[30] Ashburner M, Ball CA, Blake JA, Botstein D, Butler H, Cherry JM, Davis AP, Dolinski K, Dwight SS, Eppig JT, Harris MA, Hill DP, Issel-Tarver L, Kasarskis A, Lewis S, Matese JC, Richardson JE, Ringwald M, Rubin GM, Sherlock G 2000 Gene ontology: tool for the unification of biology. Nat Genet 25:25–29. doi:10.1038/75556. 10802651 PMC3037419

[31] Courcelle J, Khodursky A, Peter B, Brown PO, Hanawalt PC 2001 Comparative gene expression profiles following UV exposure in wild-type and SOS-deficient Escherichia coli. Genetics 158:41–64. 11333217 10.1093/genetics/158.1.41 PMC1461638

[32] Friedberg E 1985 DNA repair. WH Freeman & Co, New York, NY.

[33] Friedberg E, Walker G, Siede W 1995 DNA repair and mutagenesis, p 407–464. ASM Press, Washington, DC.

[34] Keasling J 1997 Regulation of intracellular toxic metals and other cations by hydrolysis of polyphosphate. Ann N Y Acad Sci 829:242–249. doi:10.1111/j.1749-6632.1997.tb48579.x. 9472324

[35] Ruiz ON, Alvarez D, Gonzalez-Ruiz G, Torres C 2011 Characterization of mercury bioremediation by transgenic bacteria expressing metallothionein and polyphosphate kinase. BMC Biotechnol 11:82. doi:10.1186/1472-6750-11-82. 21838857 PMC3180271

[36] Braun V 2001 Iron uptake mechanisms and their regulation in pathogenic bacteria. Int J Med Microbiol 291:67–79. doi:10.1078/1438-4221-00103. 11437341

[37] Williams KM, Rowan C, Mitchell J 2004 Effect of amine ligand bulk on the interaction of methionine with platinum (II) diamine complexes. Inorg Chem 43:1190–1196. doi:10.1021/ic035212m. 14753844

[38] Bischin C, Lupan A, Taciuc V, Silaghi-Dumitrescu R 2011 Interactions between proteins and platinum-containing anti-cancer drugs. Mini Rev Med Chem 11:214–224. doi:10.2174/138955711795049844. 21534930

[39] Dixon SJ, Stockwell BR 2014 The role of iron and reactive oxygen species in cell death. Nat Chem Biol 10:9–17. doi:10.1038/nchembio.1416. 24346035

[40] Andrews SC, Robinson AK, Rodríguez-Quiñones F 2003 Bacterial iron homeostasis. FEMS Microbiol Rev 27:215–237. doi:10.1016/S0168-6445(03)00055-X. 12829269

[41] Gardner PR, Fridovich I 1991 Superoxide sensitivity of the Escherichia coli 6-phosphogluconate dehydratase. J Biol Chem 266:1478–1483. 1846355

[42] Zheng LL, Huang CZ 2014 Selective and sensitive colorimetric detection of stringent alarmone ppGpp with Fenton-like reagent. Analyst 139:6284–6289. doi:10.1039/c4an01632g. 25315398

[43] Sarvan S, Butcher J, Stintzi A, Couture J-F 2018 Variation on a theme: investigating the structural repertoires used by ferric uptake regulators to control gene expression. Biometals 31:681–704. doi:10.1007/s10534-018-0120-8. 30014354

[44] Docampo R, de Souza W, Miranda K, Rohloff P, Moreno SN 2005 Acidocalcisomes? Conserved from bacteria to man. Nat Rev Microbiol 3:251–261. doi:10.1038/nrmicro1097. 15738951

[45] Tocheva EI, Dekas AE, McGlynn SE, Morris D, Orphan VJ, Jensen GJ 2013 Polyphosphate storage during sporulation in the gram-negative bacterium Acetonema longum. J Bacteriol 195:3940–3946. doi:10.1128/JB.00712-13. 23813732 PMC3754598

[46] Toso DB, Henstra AM, Gunsalus RP, Zhou ZH 2011 Structural, mass and elemental analyses of storage granules in methanogenic archaeal cells. Environ Microbiol 13:2587–2599. doi:10.1111/j.1462-2920.2011.02531.x. 21854518 PMC3700383

[47] Rachmilovich-Calis S, Masarwa A, Meyerstein N, Meyerstein D 2011 The effect of pyrophosphate, tripolyphosphate and ATP on the rate of the Fenton reaction. J Inorg Biochem 105:669–674. doi:10.1016/j.jinorgbio.2011.01.009. 21450270

[48] Richter Y, Fischer B 2006 Nucleotides and inorganic phosphates as potential antioxidants. J Biol Inorg Chem 11:1063–1074. doi:10.1007/s00775-006-0143-4. 16896806

[49] Biaglow JE, Kachur AV 1997 The generation of hydroxyl radicals in the reaction of molecular oxygen with polyphosphate complexes of ferrous ion. Radiat Res 148:181–187. doi:10.2307/3579576. 9254738

[50] Al-Maghrebi MA, Benov LT 2001 Polyphosphate accumulation and oxidative DNA damage in superoxide dismutase-deficient Escherichia coli. Free Radic Biol Med 31:1352–1359. doi:10.1016/s0891-5849(01)00696-7. 11728806

[51] Hawkins PT, Poyner DR, Jackson TR, Letcher AJ, Lander DA, Irvine RF 1993 Inhibition of iron-catalysed hydroxyl radical formation by inositol polyphosphates: a possible physiological function for myo-inositol hexakisphosphate. Biochem J 294:929–934. doi:10.1042/bj2940929. 8379947 PMC1134551

[52] d’Autréaux B, Pecqueur L, Gonzalez de Peredo A, Diederix RE, Caux-Thang C, Tabet L, Bersch B, Forest E, Michaud-Soret I 2007 Reversible redox-and zinc-dependent dimerization of the Escherichia coli fur protein. Biochemistry 46:1329–1342. doi:10.1021/bi061636r. 17260962

[53] Miyazawa M, Bogdan AR, Tsuji Y 2019 Perturbation of iron metabolism by cisplatin through inhibition of iron regulatory protein 2. Cell Chem Biol 26:85–97.e4. doi:10.1016/j.chembiol.2018.10.009. 30449675 PMC6338505

[54] Shiba T, Tsutsumi K, Yano H, Ihara Y, Kameda A, Tanaka K, Takahashi H, Munekata M, Rao NN, Kornberg A 1997 Inorganic polyphosphate and the induction of rpoS expression. Proc Natl Acad Sci U S A 94:11210–11215. doi:10.1073/pnas.94.21.11210. 9326588 PMC23418

[55] Morrissey JH, Choi SH, Smith SA 2012 Polyphosphate: an ancient molecule that links platelets, coagulation, and inflammation. Blood 119:5972–5979. doi:10.1182/blood-2012-03-306605. 22517894 PMC3383012

[56] Datsenko KA, Wanner BL 2000 One-step inactivation of chromosomal genes in Escherichia coli K-12 using PCR products. Proc Natl Acad Sci U S A 97:6640–6645. doi:10.1073/pnas.120163297. 10829079 PMC18686

[57] Sambrook J, Fritsch EF, Maniatis T 1989 Molecular cloning: a laboratory manual. Cold Spring Harbor Laboratory Press, Cold Spring Harbor, NY.

[58] Outten FW, Djaman O, Storz G 2004 A suf operon requirement for Fe–S cluster assembly during iron starvation in Escherichia coli. Mol Microbiol 52:861–872. doi:10.1111/j.1365-2958.2004.04025.x. 15101990

[59] Krisko A, Radman M 2013 Phenotypic and genetic consequences of protein damage. PLoS Genet 9:e1003810. doi:10.1371/journal.pgen.1003810. 24068972 PMC3778015

