# Supplementary figures and images for: Polyphosphate Functions In Vivo as an Iron Chelator and Fenton Reaction Inhibitor
Source: mBio. 2020 Jul 28;11(4):e01017-20. doi: 10.1128/mBio.01017-20 (PMC7387796; doi:10.1128/mBio.01017-20)

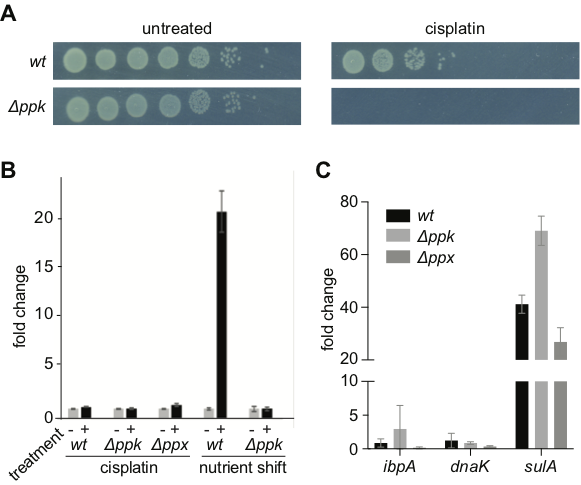

Supplement: FIG S1 [file mBio.01017-20-sf001.tif]
