# Supplementary material for: Polyphosphate Functions In Vivo as an Iron Chelator and Fenton Reaction Inhibitor
Source: mBio. 2020 Jul 28;11(4):e01017-20. doi: 10.1128/mBio.01017-20 (PMC7387796; doi:10.1128/mBio.01017-20)
Supplement: TABLE S5 [file mBio.01017-20-st005.docx]

**Supplemental Table S5: Strains, plasmids and oligos used in this study**

| **Strain name** | **Marker*** | **Relevant Genotype** | **Source** |
| --- | --- | --- | --- |
| BL21(DE3) |  | *F- ompT gal dcm lon hsdSB(rB- mB-) λ(DE3 [lacI lacUV5-T7 gene 1 ind1 sam7 nin5])* | Novagen |
| MG1655 |  | *F-, λ-, rph-1 ilvG- rfb-50* | (1) |
| MJG224 |  | MG1655 *∆ppk* | (2) |
| MJG315 |  | MG1655 *∆ppx* | (2) |
| MJG317 |  | BL21(DE3) pScPPX2 | (2) |
| FB122 | Cat | MG1655 *fur::cat* | this study |
| FB131 | Cat | MG1655 *fur::cat ∆ppk* | this study |
| FB261 | Amp | MG1655 pBAD18b | this study |
| FB262 | Amp | MG1655 *∆ppk* pBAD18b | this study |
| FB246 | Amp | MG1655 pBAD18b-*ftnA* | this study |
| FB247 | Amp | MG1655 *∆ppk* pBAD18b-*ftnA* | this study |
| FB323 | Amp | MG1655 ∆ppk pBAD18b ppkH435A | this study |
| FB345 | Kan | MG1655 pWSK129-*ppk^G688A^* | (3) |
| FB343 | Kan | MG1655 pWSK129 | (3) |
| FB264 | Amp | MG1655 pBAD18b-*ppk* | (2) |
| **Plasmids** | **Marker** | **description** | **Source** |
| pBAD18b | Amp | cloning vector with arabinose-inducible promoter | (4) |
| pKD46 | Amp | λ Red recombinase | (5) |
| pCP20 | Amp | Flp recombinase | (5) |
| pKD3 | Cat | Chloramphenicol resistance cassette donor | (5) |
| pKD4 | Kan | Kanamycin resistance cassette donor | (5) |
| pBAD18b*ftnA* | Amp | *ftnA* arabinose inducible expressing vector | this study |
| pBAD18b*ppk* | Amp | *ppk* arabinose inducible expression vector | (2) |
| pBAD18b*ppkH435A* | Amp | *ppkH435A* arabinose inducible expression vector | this study |
| pScPPX2 | Amp | ScPPX in pET-15b for protein purification | (2) |
| pWSK129 | Kan |  | (3) |
| pWSK129*ppkG688A* | Kan | ppkG688A under native *ppk* promoter | (3) |
| **Oligos** | **sequence** | | |
| ftnA_for_ pBAD18b | tcGGATCCctttgtggagcactatcatgc | | |
| ftnA_rev_pBAD18b | tcAAGCTTgtaagatttgccgtcactga | | |
| ppk_lambdared_for | atgGGTCAGGAAAAGCTATACATCGAAAAAGAGCTCGTGTAGGCTGGAGCTGCTTC | | |
| ppk_lambdared_rev | ttaTTCAGGTTGTTCGAGTGATTTGATGTAGTCATACATATGAATATCCTCCTTA | | |
| ppk_for | CGTAATTAAAGCGCCAGCTC | | |
| ppK_rev | ATCTGCATGGCACCATCTAC | | |
| fur_for | GTAAATGTAAGCTGTGCCAC | | |
| fur_rev | AGTGAGAGCTGTAACTCTCG | | |
| fur_lambdared_for | atgACTGATAACAATACCGCCCTAAAGAAAGCTGGCGTGTAGGCTGGAGCTGCTTC | | |
| fur_lambdared_rev | ttaTTTGCCTTCGTGCGCATGTTCATCTTCGCGGCACATATGAATATCCTCCTTA | | |
| pBAD_for | ctgtttctccatacccgtt | | |
| pBAD_rev | GGCTGAAAATCTTCTCTCAT | | |
| ppK_H435A_for | TGCGCCGGGGCTGAAAATTgcCGCCAAACTGTTCCTGATT | | |
| ppK_H435A_rev | AATCAGGAACAGTTTGGCGgcAATTTTCAGCCCCGGCGCA | | |

* Cat, Chloramphenicol ; Amp, ampicillin ; Kan, Kanamycin

**REFERENCES**

1. Blattner FR, Plunkett G, Bloch CA, Perna NT, Burland V, Riley M, Collado-Vides J, Glasner JD, Rode CK, Mayhew GF. 1997. The complete genome sequence of Escherichia coli K-12. science 277:1453-1462.

2. Gray MJ, Wholey W-Y, Wagner NO, Cremers CM, Mueller-Schickert A, Hock NT, Krieger AG, Smith EM, Bender RA, Bardwell JC. 2014. Polyphosphate is a primordial chaperone. Molecular cell 53:689-699.

3. Rudat AK, Pokhrel A, Green TJ, Gray MJ. 2018. Mutations in Escherichia coli polyphosphate kinase that lead to dramatically increased in vivo polyphosphate levels. Journal of bacteriology 200:e00697-17.

4. Guzman L-M, Belin D, Carson MJ, Beckwith J. 1995. Tight regulation, modulation, and high-level expression by vectors containing the arabinose PBAD promoter. Journal of bacteriology 177:4121-4130.

5. Datsenko KA, Wanner BL. 2000. One-step inactivation of chromosomal genes in Escherichia coli K-12 using PCR products. Proceedings of the National Academy of Sciences 97:6640-6645.
