# Supplementary material for: Polyphosphate Functions In Vivo as an Iron Chelator and Fenton Reaction Inhibitor
Source: mBio. 2020 Jul 28;11(4):e01017-20. doi: 10.1128/mBio.01017-20 (PMC7387796; doi:10.1128/mBio.01017-20)
Supplement: TEXT S1 [file mBio.01017-20-s0001.docx]

**Supplemental information**

**Supplemental Material and Methods:**

**Quantification of bacterial polyP levels**. PolyP measurement were performed as described in (1). Briefly, 2 ml of *E. coli* wild-type, *ppk* and *ppx* deletion cells were exponentially grown in MOPS-G and treated with 10 μg/ml cisplatin for 1 hr. As a positive control, polyP levels were tested upon nutrient shift (2). For the nutrient shift experiments, wild-type and *Δppk* strains were grown in LB until OD_600_ of 0.5 was reached. Then, cells were washed with MOPS-G low phosphate (0.132 mM K_2_HPO_4_) and resuspended in MOPS-G low phosphate for 3h at 37°C. Before and after the treatment, 1 ml of cells were harvested, resuspended in 250 μl of GITC lysis buffer (4 M guanidine isothiocyanate, 50 mM Tris-HCl, pH 7), and incubated at 95°C for 10 min. The cell lysates were supplemented with 250 µl of 95% v/v ethanol, vortexed and applied to a silica membrane spin column (Fisher scientific). Next, 5 µl of the GITC-cell lysates were collected to measure protein concentration (3), using a bovine serum albumin solution in GITC as standard. The spin columns were centrifuged for 30 sec at 16,100 x g, washed with 5 mM Tris-HCl (pH 7.5), 50 mM NaCl, 5 mM EDTA, 50% v/v ethanol, and spun down again. The bound material was eluted with 50 μl 50 mM Tris-HCl (pH 8). The extracted polyP was then digested into inorganic phosphate (P_i_) using the polyP-specific exopolyphosphatase from yeast (i.e., ScPPX) for 15 min at 37°C. P_i_ levels were quantified with an ascorbic acid-based colorimetric assay (4). The absorbance at 882 nm was normalized according to protein concentration. The fold change in polyP levels was calculated relative to the untreated sample.

**Purification of ScPPX.** ScPPX was purified according to Pokhrel et al., 2019. Briefly, cells carrying the plasmid pScPPX2 were grown at 37°C to an OD_600_ of 0.4 - 0.5. Then, 1 mM isopropyl β-D-1-thiogalactopyranoside (IPTG) was added, and the cells were incubated for another 4 h at 37°C with shaking at 180 rpm. The cells were spun down for 20 min at 5,000 x g at 4°C. The cell pellet was resuspended in 50 mM HEPES, 0.5 M NaCl, and 5 mM imidazole (pH 8.0), and incubated on ice with 1 mg /ml lysozyme, 2 mM MgCl_2_, and 50 units/ml of RNA- and DNA-degrading endonuclease (Sigma). The cells were lysed by sonication (2 cycles, 5s pulse 5s off, 2 min on ice between cycles) and the debris was spun down at 20,000 x g, 4°C for 20 min. The supernatant was filtered through a 0.8 μm pore size cellulose acetate syringe filter (Nalgene Nunc). The cell lysate was loaded onto a nickel-charged chelating column (GE Healthcare), rinsed first with 50 ml 50 mM HEPES, 0.5 M NaCl, 5 mM imidazole (pH 8.0) followed by a washing step with 50 ml of 50 mM HEPES, 0.5 M NaCl, 20 mM imidazole (pH 8.0). ScPPX was eluted with 50 mM HEPES, 0.5 M NaCl, 0.5 M imidazole (pH 8.0) and the protein concentration was measured by Bradford protein assay (3). The ScPPX protein solution dialyzed against storage buffer (20 mM Tris-HCl (pH 7.5), 50 mM KCl, 10% v/v glycerol). Finally, the protein concentration was adjusted to 1 mg/ml with storage buffer, supplemented with 0.1% w/v bovine serum albumine for stabilization and stored at 4°C for up to 6 months.

**qRT–PCR.** Gene expression analysis by real-time polymerase chain reaction (RT–PCR) was performed upon cisplatin treatment in wild-type and *Δppk* strains as described in (5). Briefly, cells were grown in MOPS-G at 37°C until OD_600_ of 0.5 was reached, and treated with or without 20 μg/ml cisplatin for 15 minutes. Next, cells were spun down and resuspended in 1 ml of ice-cold methanol (– 80°C) to stop transcription. The RNA was extracted using the NucleoSpin RNA kit (Macherey & Nagel) and DNA-free kit (Ambion). A PrimeScript 1st strand cDNA Synthesis Kit (Takara) was used to generate cDNA, and RT–PCRs were set up with SYBR GreenER qRT–PCR mix (Invitrogen) and a Mastercycler ep realplex real-time PCR system (Eppendorf). Expression ratios were calculated from the comparison with the expression of each gene in untreated cultures by the ΔΔCT method and normalized to the expression of rrsD (encoding 16S rRNA), the expression of which did not change under the conditions tested. Primers used: 16S_RT_for: AGAGTTTGATCCTGGCTCAG; 17S- RT_rev: TTACTCACCC GTCCGCCACTC; IbpA_RT_for: TGCTATTGGATTTGACCGTTTG; ibpA_RT_rev: CGGCAC GTTATACGGAGGGTAGCCG; dnaK_RT_for:ACAGCACCCGTAA GCAGGTTGAAGAA, dnaK_RT_rev: TGGGCGATTTCCATCAGTTTCTGGGA; sulA_RT_for: CGGGCTTATCAGTG AAGTTGTCTAT, SulA_RT_rev: CTGGCTAATCTGCATTACTTTCGTT.

**Supplemental References:**

1. Pokhrel A, Lingo JC, Wolschendorf F, Gray MJ. 2019. Assaying for inorganic polyphosphate in bacteria. JoVE (Journal of Visualized Experiments):e58818.

2. Ault-Riché D, Fraley CD, Tzeng C-M, Kornberg A. 1998. Novel assay reveals multiple pathways regulating stress-induced accumulations of inorganic polyphosphate in Escherichia coli. Journal of bacteriology 180:1841-1847.

3. Bradford MM. 1976. A rapid and sensitive method for the quantitation of microgram quantities of protein utilizing the principle of protein-dye binding. Analytical biochemistry 72:248-254.

4. Christ JJ, Blank LM. 2018. Enzymatic quantification and length determination of polyphosphate down to a chain length of two. Analytical biochemistry 548:82-90.

5. Dahl J-U, Gray MJ, Bazopoulou D, Beaufay F, Lempart J, Koenigsknecht MJ, Wang Y, Baker JR, Hasler WL, Young VB. 2017. The anti-inflammatory drug mesalamine targets bacterial polyphosphate accumulation. Nature microbiology 2:1-5.
